# Paradoxical clinical outcomes of severe versus very severe aortic valve stenosis after transcatheter aortic valve implantation? a propensity score matched analysis and review of literature

**DOI:** 10.1016/j.ijcha.2025.101710

**Published:** 2025-06-02

**Authors:** Matthias Hammerer, Elke Boxhammer, Erika Prinz, Bernhard Scharinger, Wilfried Wintersteller, Uta C. Hoppe

**Affiliations:** a Department of Internal Medicine II, Division of Cardiology, Austria; b Department of Radiology Paracelsus Medical University of Salzburg Müllner Hauptstraße 48, 5020 Salzburg, Austria

**Keywords:** Severe aortic valve stenosis, Very severe aortic valve stenosis, Transcatheter aortic valve implantation, Clinical outcomes, Mortality

## Abstract

**Background:**

Very severe aortic stenosis (VSAS) is a critical condition with unfavourable clinical outcomes if left untreated or treated by surgical valve replacement. In contrast, after transcatheter valve implantation (TAVI) outcomes seem to be similar or – paradoxically – even better compared to severe aortic stenosis (SAS), as indicated by previous studies.

**Methods:**

Data of patients from a single centre who underwent TAVI were retrospectively analysed. Patients with concordant AS (n = 475) were divided into SAS (n = 379) and VSAS (n = 96) groups. These groups are compared in terms of procedural (safety) and long-term (efficacy) outcomes, using propensity score matching. In addition, a review of relevant literature is provided.

**Results:**

After propensity score matching, 96 patients remained in each group. Procedural outcomes did not differ significantly between VSAS and SAS groups. Cox proportional hazards regression analysis showed a favourable trend toward lower overall mortality within a mean follow-up of 42 months after TAVI in the VSAS group (hazard ratio, HR, 0.668; 95 % confidence interval, CI, 0.430–1.038). This difference did not reach statistical significance (p = 0.073), however, it was significant in the subgroups of females (p = 0.045) and patients with NYHA class III (p = 0.043).

**Conclusion:**

Our analysis confirms – in line with previous studies – that patients with VSAS represent a substantial subgroup and have at least as favourable or – paradoxically −even better clinical results after TAVI compared to patients with SAS. Therefore, TAVI should not be withheld from these patients.

## Graphical abstract

**Figure:**
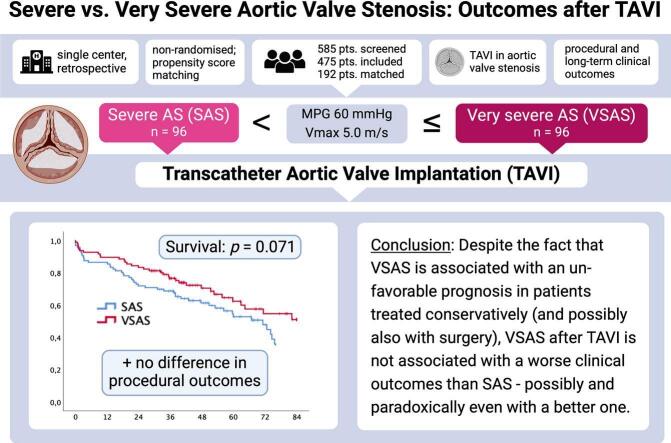

## 1 Introduction

Aortic stenosis (AS) represents the most common valvular heart disease in older adults, characterized by progressive calcific narrowing of the aortic valve and resulting in pressure overload, heart failure symptoms, and increased mortality. Current clinical guidelines [1,2] classify AS severity based on hemodynamic parameters, with severe AS (SAS) defined by a peak transvalvular velocity (Vmax) > 4.0 m/s and mean pressure gradient (MPG) > 40 mmHg in transthoracic echocardiography (TTE). Within this spectrum, a subset of patients meeting higher thresholds (Vmax ≥ 5.0 m/s, MPG ≥ 60 mmHg) has been designated as having very severe aortic stenosis (VSAS) — a phenotype associated with markedly poor outcomes under conservative or delayed management, even in the absence of symptoms [[3], [4], [5], [6], [7], [8]]. While both SAS and VSAS represent a high-risk condition with increased mortality if left untreated, the prognostic nuances between these groups after transcatheter or surgical valve replacement remain underexplored.

Transcatheter aortic valve implantation (TAVI) has redefined therapeutic strategies for AS, offering a minimally invasive alternative to surgical aortic valve replacement (SAVR) with comparable outcomes, even in lower-risk populations [[9], [10], [11]]. Intriguingly, several retrospective studies suggest that patients with VSAS may exhibit similar [[12], [13], [14], [15]] — or even more favourable [16] — clinical outcomes after TAVI compared to those with SAS, despite a higher hemodynamic burden. These findings are unexpected and poorly understood, given that higher transvalvular gradients typically correlate with advanced disease. Limitations in previous research include small sample sizes, methodological heterogeneity, and limited risk adjustment, making it difficult to discern whether the observed paradox reflects pathophysiological phenomena or selection biases.

To address this knowledge gap, we conducted a propensity score–matched analysis comparing procedural safety and long-term survival outcomes between patients with SAS and VSAS undergoing transfemoral TAVI at a single academic center. Our study aims to clarify whether VSAS confers differential prognostic implications in the TAVI population and to provide a nuanced understanding of this high-risk subgroup, while accounting for confounding baseline characteristics. In addition, we provide a review of relevant literature.

## 2 Methods

### 2.1 Study cohort

**Fig. 1:**
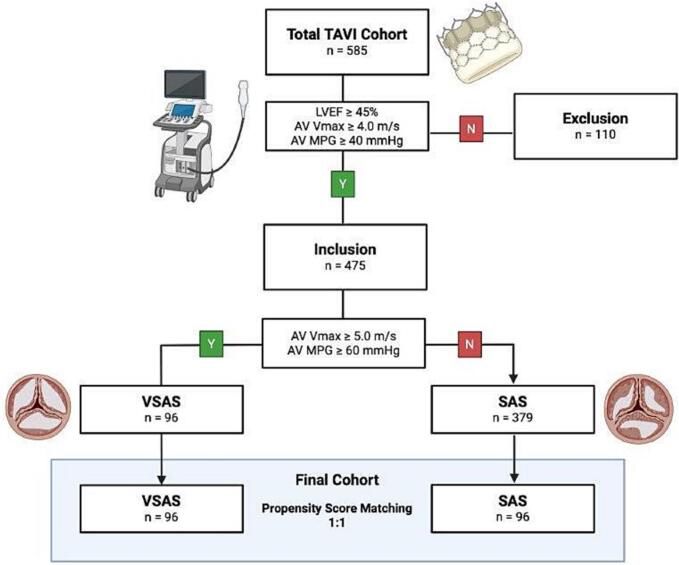
**Study flow chart.** TAVI, transcatheter aortic valve implantation; SAS, severe aortic valve stenosis; VSAS, very severe aortic valve stenosis; LVEF, left ventricular ejection fraction; AV Vmax, aortic valve maximal systolic transvalvular flow velocity; AV MPG, aortic valve mean systolic pressure gradient.

This research analysed a consecutively enrolled cohort of 585 patients considered suitable for TAVI at Paracelsus Medical University Hospital in Salzburg between 2016 and 2022. Eligibility for TAVI was determined by a multidisciplinary heart team comprising cardiac surgeons, interventional cardiologists, and anesthesiologists. Patients with discordant AS based on echocardiographic criteria including left ventricular ejection fraction (LVEF) below 50 %, AV Vmax < 4.0 m/s, or an AV MPG < 40 mmHg (i.e. low-flow low-gradient or paradoxical low-flow low-gradient stenosis, not allowing for a clear differentiation between SAS and VSAS) were excluded from further analyses. The remaining patients with concordant AS were further divided into SAS and VSAS groups, and a propensity score matching process was conducted on a 1:1 basis (See Fig. 1).

### 2.2 Ethical considerations

The study received authorization from the Ethics Committee of the State of Salzburg (approval number EK-Nr. 1082/2024). All research procedures adhered to ethical standards outlined in the Declaration of Helsinki and the principles of Good Clinical Practice (ICH-GCP). Due to the study's retrospective design, the Ethics Committee waived the need for obtaining written informed consent from participants.

### 2.3 Data collection

Patient information was obtained from the ORBIS electronic medical records platform (Agfa Healthcare, Version 08043301.04110DACHL) and an additional medical archiving system (Krankengeschichtsarchiv System, Uniklinikum Salzburg, Softworx by Andreas Schwab TM, 2008). Data collected encompassed patient charts, summaries of hospital admissions and discharges, as well as echocardiographic and other imaging reports relevant to the TAVI procedure.

### 2.4 Transthoracic echocardiography

Standard transthoracic echocardiograms were conducted 1 to 4 weeks prior to the TAVI procedure, utilizing either the iE33 or Epiq 5 ultrasound systems (Philips Healthcare, Hamburg, Germany). These echocardiograms were performed by clinicians with a minimum of 4 years of dedicated experience in echocardiography. The classification of severe aortic stenosis adhered to the latest guidelines, which define severity through specific echocardiographic criteria [1,2]. The following cut-off values were utilized for classification of SAS and VSAS:

• SAS: AV Vmax ≥ 4.0 to 5.0 m/s; AV MPG ≥ 40 to 60 mmHg
• VSAS: AV Vmax ≥ 5.0 m/s; AV MPG ≥ 60 mmHg

### 2.5 Computed tomography angiography and calcium scoring

All patients underwent standard preprocedural computed tomography (CT) imaging, including ECG-gated angiography to assess vascular access and aortic root anatomy, and non-contrast CT for aortic valve calcium (AVC) quantification. Scans were performed using 128- or 256-slice dual-source CT systems (Revolution, GE Healthcare, IL, USA or Somatom Definition AS+, Siemens Healthcare, Erlangen, Germany). Annulus dimensions and AVC scores were assessed using dedicated imaging software (Impax, Agfa-Gevaert, Mortsel, Belgium; IntelliSpace, Philips, Amsterdam, Netherlands). AVC was indexed to body surface area (AVCi) and annulus area (AVCd) to support prosthesis sizing and risk stratification.

### 2.6 TAVI procedure

All 585 patients underwent transfemoral TAVI using second- or third-generation self-expanding valve systems (CoreValve™ Evolut™ R or Evolut™ Pro; Medtronic Inc., Minneapolis, MN, USA), in accordance with standardized protocols at our institution. This valve platform and a transfemoral arterial access route were uniformly used during the study period. Pre-procedural imaging, which included TTE, ECG gated CTA, and, when necessary, transesophageal echocardiography, was used for optimal procedure planning according to the manufacturer’s recommendations.

### 2.7 Outcomes

The primary outcome of this study was long-term overall survival, with a maximum follow-up of 84 months, to assess TAVI efficacy in SAS and VSAS patients, respectively.

Secondary outcomes included overall mortality within the first 30 days post-procedure as an indicator of immediate procedural safety. Additional secondary outcomes were adverse events according to the Valve Academic Research Consortium-3 (VARC-3) criteria. [17].

### 2.8 Statistical analysis

Statistical analyses were performed using SPSS software (Version 25.0, SPSS Inc., Armonk, NY, USA) and R (Version 4.2.3, R Foundation for Statistical Computing, Vienna, Austria). Visualizations were carried out using SPSS and GraphPad Prism software (GraphPad Prism version 8.0.0, GraphPad Software, San Diego, CA, USA, www.graphpad.com).

A detailed post hoc power analysis using G*Power 3.1 demonstrated that with 96 patients in each group, the study achieved a statistical power of 93 %. This high level of power reflects the study's strong ability to detect meaningful differences or significant effects between groups, reducing the likelihood of a Type II error (failing to detect an effect that truly exists). The analysis was performed with a conventional alpha (α) level of 0.05, which is the standard threshold for statistical significance, and assumed a medium effect size (d = 0.5).

A propensity score matching was applied to create balanced cohorts of patients with VSAS and SAS, aiming to minimize confounding. Matching was performed on treatment group (VSAS vs. SAS), sex, and age:

• Exact Matching by Sex: To ensure equal representation of sexes, patients were first stratified by sex, allowing only same-sex pairs between VSAS and SAS groups.
• Nearest-Neighbor Matching on Age: Within each sex-stratified group, nearest-neighbor matching was applied based on age to match VSAS and SAS patients with the closest possible age. A 1:1 matching ratio was used.

Post-matching, we confirmed balance between groups in age and sex distributions. All analyses were conducted in R using the MatchIt package.

Variable distributions were evaluated with the Kolmogorov–Smirnov–Lilliefors test. For continuous variables with normal distribution, results were reported as mean standard deviation (SD) and compared via the unpaired Student's *t*-test. Non-normally distributed variables were summarized as median with interquartile range (IQR) and analysed using the Mann–Whitney *U* test. Categorical variables were described as frequencies or percentages and compared with the chi-squared test.

Kaplan–Meier survival analysis was used to compare the long-term mortality as a primary endpoint between patients with VSAS vs. SAS, with statistical differences assessed using log-rank tests and associated risk numbers. Additionally, a separate, univariate Cox regression analysis was examined whether different baseline clinical characteristics linked to an increased risk of mortality regarding the severity of AS.

Finally, Cox regression analysis was conducted to identify clinical characteristics associated with higher probability of mortality regarding different stages of aortic stenosis severity. To ensure comparability, metric data were standardized through z-transformation. Variables with a p-value ≤ 0.100 in univariate analysis were included in a multivariable Cox regression model refined through backward elimination to identify independent predictors of long-term mortality in patients with VSAS vs. SAS.

In all statistical analyses, a p-value < 0.050 was considered statistically significant.

## 3 Results

### 3.1 Patient selection

The study cohort initially included a total of 585 patients who underwent TAVI. Of these, 110 patients with discordant AS were excluded. This led to a final inclusion of 475 patients with concordant AS for further analysis.

Within this included cohort, patients were further divided into SAS (n = 379) and VSAS (n = 96) groups following the aforementioned echocardiographic criteria. A propensity score matching process was conducted on a 1:1 basis to create a balanced final cohort, resulting in two matched groups: 96 patients in the VSAS group and 96 patients in the SAS group. This final cohort was used for comparative analyses (See Fig. 1).

### 3.2 Baseline characteristics

Table 1 summarizes the baseline characteristics of 192 patients, equally divided into SAS and VSAS groups. Demographics and comorbidities were broadly similar in both groups except the rates of coronary artery disease (CAD) and New York Heart Association (NYHA) class ≥ III status. CAD was significantly more common in the SAS group (61.5 %) compared to the VSAS group (45.8 %, p = 0.030), and NYHA class ≥ III status was more prevalent in the SAS group (54.2 % vs. 37.5 %, p = 0.022).

**Table 1:** Baseline characteristics.

	**Total**	**SAS**	**VSAS**	**p**
	N = 192	N = 96	N = 96	
**Demographics**				
Sex (male) — n (%)	76 (39.6)	38 (39.6)	38 (39.6)	1
Age (years) — mean ± SD	81.2 ± 4.8	81.2 ± 4.8	81.2 ± 4.9	0.905
Height (cm) — mean ± SD	166.9 ± 9.2	166.2 ± 8.6	167.6 ± 9.7	0.304
Weight (kg) — mean ± SD	72.3 ± 14.7	73.3 ± 15.4	71.2 ± 14.1	0.32
BMI (kg/m^2^) — mean ± SD	25.8 ± 4.4	26.4 ± 4.7	25.3 ± 4.0	0.092
BSA (m^2^) — mean ± SD	1.8 ± 0.3	1.8 ± 0.3	1.8 ± 0.3	0.82
Diabetes mellitus — n (%)	47 (24.5)	22 (22.9)	25 (26.0)	0.615
Arterial hypertension — n (%)	170 (88.5)	86 (89.6)	84 (87.5)	0.65
CAD — n (%)	103 (53.6)	59 (61.5)	44 (45.8)	0.03
AF — n (%)	77 (40.1)	42 (43.8)	35 (36.5)	0.303
MI before TAVI — n (%)	11 (5.7)	7 (7.3)	4 (4.2)	0.352
TIA/Stroke before TAVI — n (%)	19 (9.9)	12 (12.5)	7 (7.3)	0.227
PAOD — n (%)	24 (12.5)	9 (9.4)	15 (15.6)	0.19
COPD — n (%)	21 (10.9)	11 (11.5)	10 (10.4)	0.817
NYHA ≥ III — n (%)	88 (45.8)	52 (54.2)	36 (37.5)	0.022
**Laboratory**				
Creatinine (mg/dl) — median ± IQR	1.1 ± 0.5	1.1 ± 0.4	1.0 ± 0.7	0.973
proBNP (pg/ml) — median ± IQR	2445.5 ± 4089.0	2455.0 ± 3881.9	2436.0 ± 6322.9	0.747
Hematocrite (%) — median ± IQR	39.1 ± 5.8	40.1 ± 4.5	37.9 ± 8.5	0.577
Hemoglobin (g/dl) — median ± IQR	13.3 ± 1.9	13.4 ± 1.4	13.1 ± 2.7	0.931
CK (U/l) — median ± IQR	76.5 ± 46.3	77.0 ± 50.0	70.0 ± 50.0	0.489
**Echocardiography**				
LVEF (%) — median ± IQR	55.0 ± 8.0	55.0 ± 6.8	55.0 ± 8.0	0.198
SVi (ml/m^2^) — median ± IQR	48.3 ± 22.5	46.3 ± 23.4	49.3 ± 21.8	0.299
IVSd (mm) — median ± IQR	13.0 ± 1.8	13.0 ± 2.5	14.0 ± 2.0	0.017
LVEDD (mm) — median ± IQR	46.0 ± 7.5	46.0 ± 6.5	45.0 ± 7.0	0.594
AV Vmax (m/s) — median ± IQR	4.6 ± 0.7	4.4 ± 0.3	5.0 ± 0.3	< 0.001
AV MPG (mmHg) median ± IQR	52.5 ± 18.4	44.0 ± 7.0	61.9 ± 9.0	< 0.001
Aortic regurgitation ≥ II° — n (%)	37 (19.3)	18 (18.8)	19 (19.8)	0.855
Mitral regurgitation ≥ II° — n (%)	65 (33.9)	37 (38.5)	28 (29.2)	0.17
Tricuspid regurgitation ≥ II° — n (%)	50 (26.0)	29 (30.2)	21 (21.9)	0.188
**Computed Tomography**				
Annulus diameter (mm) — median ± IQR	25.5 ± 3.4	25.5 ± 3.5	25.5 ± 3.5	0.74
Annulus perimeter (mm) — median ± IQR	78.0 ± 10.8	78.0 ± 11.5	78.0 ± 12.0	0.943
Annulus area (cm^2^) — median ± IQR	4.8 ± 1.4	4.9 ± 1.5	4.8 ± 1.4	0.594
AVC (AU) — median ± IQR	4152.0 ± 3207.0	3617.0 ± 2584.5	5397.0 ± 3692.0	< 0.001
AVCi (AU/m^2^) — median ± IQR	2167.0 ± 1490.9	2077.3 ± 1252.3	2487.1 ± 1512.0	< 0.001
AVCd (AU/cm^2^) — median ± IQR	789.1 ± 501.5	729.2 ± 382.5	960.3 ± 540.8	< 0.001

SAS, severe aortic valve stenosis; VSAS, very severe aortic valve stenosis; BMI, body mass index; BSA, body surface area; CAD, coronary artery disease; AF, atrial fibrillation; MI, myocardial infarction; TAVI, transcatheter aortic valve implantation; TIA, transient ischemic attack; PAOD, peripheral arterial occlusive disease; COPD, chronic obstructive pulmonary disease; NYHA, New York Heart Association; proBNP, pro brain natriuretic peptide; CK, creatine kinase; LVEF, left ventricular ejection fraction; SVi, stroke volume index; IVSd, interventricular septum diastolic thickness; LVEDD, left ventricular enddiastolic diameter; AV Vmax, aortic valve maximal systolic transvalvular flow velocity; AV MPG, aortic valve mean systolic pressure gradient; AVC, aortic valve calcium; AU, Agatston units; AVCi, AVC indexed to body surface area; AVCd, AVC indexed to aortic valve annulus area; SD, standard deviation; IQR, interquartile range.

By definition, echocardiographic criteria — such as AV Vmax, AV MPG — were significantly higher in the VSAS group, with no significant differences in LVEF and stroke volume indexed to body surface area (SVi). Additionally, CT imaging revealed a higher burden of aortic valve calcification in VSAS patients (p < 0.001 for AVC score, AVCi and AVCd), while annulus dimensions were similar between groups.

### 3.3 Primary endpoint: Kaplan-Meier curve and Cox regression analysis of long-term mortality

**Fig. 2:**
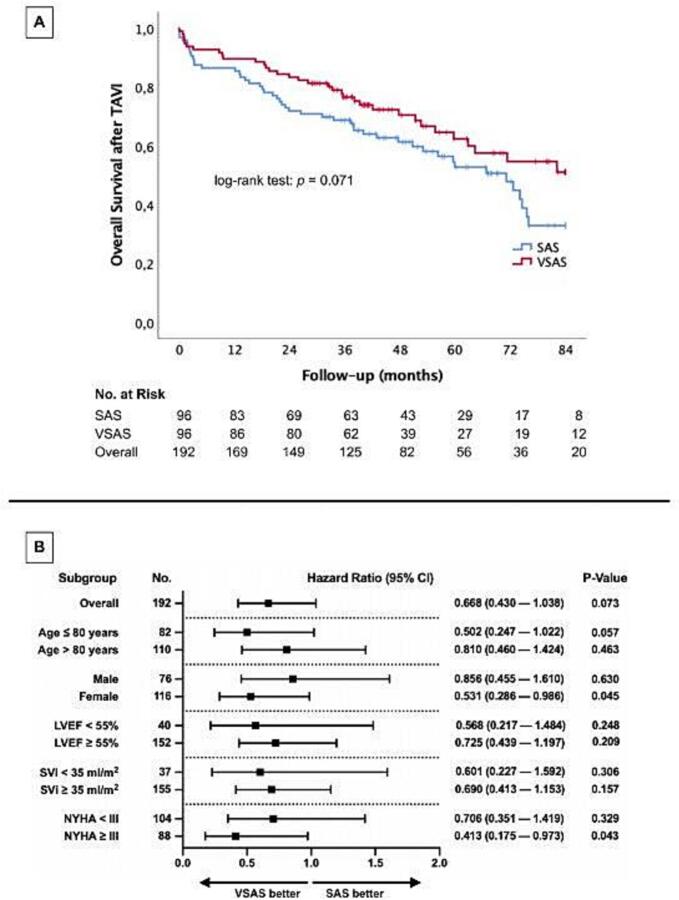
**A: Kaplan-Meier curves for overall survival after TAVI; B: Cox proportional hazards regression analysis for overall mortality across several subgroups.** SAS, severe aortic valve stenosis; VSAS, very severe aortic valve stenosis; TAVI, transcatheter aortic valve implantation; LVEF, left ventricular ejection fraction; SVi, stroke volume index; NYHA, New York Heart Association; 95% CI, 95% confidence interval.

Fig. 2A shows Kaplan-Meier survival curves comparing overall mortality after TAVI between patients with SAS and VSAS. The VSAS group (red line) demonstrates a trend towards improved overall survival compared to the SAS group (blue line), which did not reach statistical significance (log-rank test: p = 0.071). Mean follow-up time was 42.4 months (IQR 37.8 months).

Fig. 2B presents a Cox proportional hazards regression analysis, evaluating HR for overall mortality across several subgroups. The overall Hazard Ratio (HR) for VSAS relative to SAS is 0.668 (95 % CI: 0.430–1.038), with a p-value of 0.073, indicating a favorable trend toward lower mortality in the VSAS group. Importantly, significant mortality reductions are observed in certain subgroups: females in the VSAS group show a significantly lower mortality risk with a HR of 0.531 (95 % CI: 0.286–0.986; p = 0.045), and patients with NYHA class III or higher also experience a significant survival benefit with a HR of 0.413 (95 % CI: 0.175–0.973; p = 0.043).

### 3.4 Cox regression analysis: Predictors of overall mortality in SAS vs. VSAS

Table 3 presents results from a Cox regression analysis identifying predictors of overall mortality in patients with SAS and VSAS. Both univariate and multivariable analyses were conducted.

In the SAS group, a relatively low LVEF (within the normal range of >50 %) was associated with higher mortality in both univariate and multivariable analyses (univariate HR: 0.691, 95 % confidence interval, CI: 0.514–0.930, p = 0.015; multivariable HR: 0.695, 95 % CI: 0.499–0.968, p = 0.032). For VSAS patients, prior myocardial infarction was a significant predictor of mortality (univariate HR: 4.485, 95 % CI: 1.570–12.809, p = 0.005; multivariable HR: 8.071, 95 % CI: 2.246–29.003, p = 0.001).

### 3.5 Secondary endpoint: procedural data and short term outcomes

Table 2 outlines the procedural data and clinical outcomes. Pre-dilatation was performed in only a small fraction of cases (3.1 %) and was not significantly more frequent in VSAS than in SAS (5.2 % vs. 1.0 %, p = 0.097). Post-dilatation, however, was significantly more common in the VSAS group compared to the SAS group (24.0 % vs. 10.4 %, p = 0.013).

**Table 2:** Procedural TAVI data and outcomes, clinical 30-day outcomes.

	**Total**	**SAS**	**VSAS**	**p**
	N = 192	N = 96	N = 96	
**Procedural data**				
Transfemoral approach — n (%)	192 (100.0)	96 (100.0)	96 (100.0)	−
Medtronic CoreValve series — n (%)	192 (100.0)	96 (100.0)	96 (100.0)	−
Pre-dilatation — n (%)	6 (3.1)	1 (1.0)	5 (5.2)	0.097
Post-dilatation — n (%)	33 (17.2)	10 (10.4)	23 (24.0)	0.013
**Procedural complications**				
Cardiac tamponade — n (%)	2 (1.0)	1 (1.0)	1 (1.0)	−
Cardiogenic shock — n (%)	2 (1.0)	2 (2.1)	0 (0.0)	0.155
Septic shock — n (%)	1 (0.5)	0 (0.0)	1 (1.0)	0.316
Conversion to open-chest surgery — n (%)	1 (0.5)	0 (0.0)	1 (1.0)	0.316
Valve migration	1 (0.5)	0 (0.0)	1 (1.0)	0.316
Transfusion of ≥ 4 units of blood	2 (1.0)	1 (1.0)	1 (1.0)	−
Myocardial infarction	0 (0.0)	0 (0.0)	0 (0.0)	−
CABG	0 (0.0)	0 (0.0)	0 (0.0)	−
Aortic dissection	0 (0.0)	0 (0.0)	0 (0.0)	−
**Clinical outcomes**				
30-day death — n (%)	7 (3.6)	4 (4.2)	3 (3.1)	0.7
30-day stroke/TIA — n (%)	7 (3.6)	3 (3.1)	4 (4.2)	0.7
30-day pacemaker necessity — n (%)	27 (14.1)	11 (11.5)	16 (16.7)	0.299
30-day major vascular complications/bleedings — n (%)	9 (4.7)	4 (4.2)	5 (5.2)	0.733
Moderate PVL — n (%)				
	31 (16.1)	17 (17.7)	14 (14.6)	0.556

TAVI, transcatheter aortic valve implantation; SAS, severe aortic valve stenosis; VSAS, very severe aortic valve stenosis; CABG, coronary artery bypass graft; TIA, transient ischemic attack; PVL; paravalvular leakage.

Procedural complications were rare across both groups, with low incidences of cardiac tamponade, cardiogenic and septic shock as well as conversion to open-chest surgery or valve migration. Other severe complications such as myocardial infarction or aortic dissection were not observed. In terms of clinical outcomes, the 30-day mortality rate was low (3.6 %) and similar in both groups. Other 30-day outcomes, including stroke/transient ischemic attack (3.6 %), need for a permanent pacemaker (14.1 %), and major vascular complications or bleeding (4.7 %), did not significantly differ between SAS and VSAS patients. Moderate paravalvular leakage (PVL) was observed in 16.1 % of patients, with a similar incidence across both groups.

### 3.6 Possible high-risk criteria in patients with severe aortic stenosis: Low normal LVEF, low SVi, high AVC scores

Within the SAS group, 24 %, 35 %, and 38 % of patients had low normal LVEF (≥50 % and < 55 %), low SVi (<35 ml/min/m^2^) and high AVC scores (>3700 for men or > 2400 for women), respectively, the latter indicating VSAS as our study group recently proposed. [18] However, there was relatively little overlap between these presumed high-risk criteria, with only 3 % percent of patients fulfilling all three of the above criteria, and only 6 %, 8 %, and 9 % meeting two of these criteria (LVEF plus SVi; SVi plus AVC; AVC plus LVEF; respectively). Due to the low numbers of patients in these subsets, further analyses regarding prognostic significance had to be omitted.

## 4 Discussion

As the main result of our analysis patients with VSAS showed a non-inferior survival after TAVI compared to patients with SAS in this propensity score-matched TAVI population – despite having significantly higher AVC scores and higher rates of post-dilatation during TAVI procedure. In fact, survival in the VSAS group tends to be even better than in the SAS group, although this did not reach statistical significance. However, Cox regression analysis showed female patients and patients with NYHA class III symptoms to benefit significantly from TAVI (Fig. 2), whereas low LVEF and previous myocardial infarction emerged as independent predictors of mortality in SAS and VSAS, respectively (Table 3). Our study thus confirms the overall trends shown by several previous studies.

**Table 3:** Univariate and multivariable Cox regression analysis.

**Cox Regression Analysis**	**Univariate**		**Multivariable**	
	**Hazard Ratio (95 % CI)**	**p-value**	**Hazard Ratio (95 % CI)**	**p-value**
**Overall mortality − SAS**				
Age	0.887 (0.655–1.202)	0.439		
Sex (female)	0.725 (0.410–1.284)	0.270		
BMI	1.232 (0.928–1.636)	0.149		
BSA	1.221 (0.885–1.685)	0.223		
NYHA ≥ III	1.059 (0.540–2.077)	0.869		
Diabetes mellituis	1.317 (0.693–2.500)	0.400		
Arterial hypertension	0.515 (0.229–1.157)	0.108		
CAD	0.643 (0.364–1.138)	0.130		
AF	1.052 (0.594–1.863)	0.861		
MI before TAVI	1.306 (0.466–3.661)	0.612		
TIA/Stroke before TAVI	0.631 (0.249–1.601)	0.332		
PAOD	0.966 (0.345–2.709)	0.948		
COPD	0.746 (0.295–1.886)	0.536		
Creatinine	1.037 (0.849–1.267)	0.721		
proBNP	1.257 (0.864–1.830)	0.232		
Hematocrit	1.126 (0.927–1.367)	0.233		
Hemoglobin	0.970 (0.730–1.291)	0.837		
Creatine kinase	0.702 (0.443–1.112)	0.132		
LVEF	0.691 (0.514–0.930)	0.015	0.695 (0.499–0.968)	0.032
SVi	0.900 (0.661–1.228)	0.507		
IVSd	1.049 (0.781–1.410)	0.749		
LVEDD	1.186 (0.951–1.480)	0.130		
Aortic regurgitation ≥ II°	0.751 (0.351–1.609)	0.462		
Mitral regurgitation ≥ II°	1.524 (0.864–2.687)	0.145		
Tricuspid regurgitation ≥ II°	0.947 (0.513–1.745)	0.860		
AVC	1.306 (0.972–1.755)	0.076	1.181 (0.855–2.630)	0.313
AVCi	1.267 (0.921–1.742)	0.146		
AVCd	1.198 (0.895–1.603)	0.225		
**Overall mortality − VSAS**				
Age	1.161 (0.829–1.626)	0.385		
Sex (female)	0.454 (0.231–0.893)	0.022	0.460 (0.197–1.071)	0.072
BMI	1.168 (0.835–1.634)	0.364		
BSA	1.313 (0.893–1.930)	0.167		
NYHA ≥ III	0.520 (0.213–1.272)	0.152		
Diabetes mellituis	1.237 (0.601–2.547)	0.563		
Arterial hypertension	0.796 (0.307–2.063)	0.639		
CAD	0.934 (0.476–1.835)	0.843		
AF	1.594 (0.807–3.146)	0.179		
MI before TAVI	4.485 (1.570–12.809)	0.005	8.071 (2.246–29.003)	0.001
TIA/Stroke before TAVI	1.175 (0.356–3.876)	0.791		
PAOD	0.966 (0.345–2.709)	0.948		
COPD	1.425 (0.550–3.696)	0.466		
Creatinine	1.114 (0.913–1.361)	0.288		
proBNP	0.724 (0.268–1.954)	0.524		
Hematocrit	0.946 (0.685–1.308)	0.739		
Hemoglobin	0.953 (0.692–1.314)	0.770		
Creatine kinase	0.795 (0.549–1.150)	0.222		
LVEF	0.994 (0.683–1.444)	0.973		
SVi	0.703 (0.485–1.020)	0.064	0.616 (0.378–1.004)	0.052
IVSD	1.301 (0.912–1.855)	0.147		
LVEDD	0.699 (0.232–2.103)	0.524		
Aortic regurgitation ≥ II°	1.403 (0.603–3.241)	0.427		
Mitral regurgitation ≥ II°	0.923 (0.441–1.933)	0.832		
Tricuspid regurgitation ≥ II°	1.333 (0.621–2.862)	0.460		
AVC	1.350 (0.940–1.937)	0.104		
AVCi	1.381 (0.965–1.977)	0.077	1.046 (0.487–2.247)	0.908
AVCd	1.535 (1.015–2.321)	0.042	1.319(0.839–2.073)	0.231

SAS, severe aortic valve stenosis; VSAS, very severe aortic valve stenosis; BMI, body mass index; BSA, body surface area; NYHA, New York Heart Association; CAD, coronary artery disease; AF, atrial fibrillation; MI, myocardial infarction; TAVI, transcatheter aortic valve implantation; TIA, transient ischemic attack; PAOD, peripheral arterial occlusive disease; COPD, chronic obstructive pulmonary disease; proBNP, pro brain natriuretic peptide; LVEF, left ventricular ejection fraction; SVi, stroke volume index; IVSd, interventricular septum diastolic thickness; LVEDD, left ventricular enddiastolic diameter; AVC, aortic valve calcium; AU, Agatston units; AVCi, AVC indexed to body surface area; AVCd, AVC indexed to aortic valve annulus area; 95% CI, 95% confidence interval.

Although we included patients consecutively, our study population only consists of patients treated with second- or third-generation self-expanding CoreValve™ systems via transfemoral access, which was the standardized approach at our exclusively non-surgical centre throughout the study period. However, this enhances comparability within the cohort and reduces confounding due to device- or access-related variability. Such an approach was even supported by a recent review emphasizing uniformity in valve type and procedural access to improve the interpretability of TAVI outcome studies [19]. Since the vast majority of all TAVIs (90–95 %) are now performed via the femoral approach in accordance with guidelines, we believe that the confinement to the femoral access does not negatively affect the applicability of our study to the vast majority of everyday patients.

### 4.1 Review of literature and comparison to our results

A literature search in the Pubmed database revealed five studies on this topic. An overview is shown in Table 4.. All of these are single-center studies of moderate sizes and – with one exception [12] – retrospective design:

• In the study by *Onada et al.* [16] VSAS patients (n = 65) were significantly less at risk (p = 0.01) to reach the primary endpoint (all-cause death, hospitalization for heart failure) than SAS patients (n = 174) during a follow-up period of three years after TAVI. This seemingly “paradoxical” result was driven by a reduction of heart failure re-hospitalization rates (p = 0.047). The effect remained significant after adjustment for potential confounders (p = 0.01). Pre-/post-dilatation rates and procedural safety endpoints did not differ significantly.
• *Angellilis et al.* [12] compared prospectively clinical outcomes after TAVI in SAS (n = 535) and VSAS patients (n = 102) and found no significant difference for the primary endpoint of all-cause mortality (0.0 % vs. 1.0 %; p = 0.16) during a short-term follow-up of 30 days. The cumulative Kaplan Meier curves showed a significant lower rate of the secondary endpoint of cardiac rehospitalization in the VSAS group up to 12 months after TAVI (p = 0.036), but no difference in survival rates (p = 0.097). During TAVI procedure patients with SAS needed less pre- and/or post-dilatation than patients with VSAS (49.2 % vs. 57.8 %; p = 0.002; and 32.4 % vs. 58.8 %, p < 0.001, respectively), other procedural endpoints did not differ significantly. As additional secondary endpoints, this study examined the prevalence of concentric and excentric left ventricular hypertrophy, which showed a more pronounced improvement 30 days after TAVI in the VSAS group compared to SAS.
• *Kobayashi et al.* [13] found no significant difference for a combined safety- and efficacy-endpoint (all-cause mortaility, stroke, rehospitalization for heart failure, major bleeding; 20.3 vs. 16.9 %; p = 0.365) during a follow-up of 12 months after TAVI in patients with SAS (n = 215) and VSAS (n = 83). The endpoint components and other procedural complications did not differ significantly. Pre- and post-dilatation rates were not specified.
• *Saji et al.* [14] analyzed SAS (n = 363) versus VSAS (n = 258) and added “extreme severe” AS (Vmax ≥ 6.0 m/s) as a new subgroup of patients with even more advanced AS (n = 62). They found no significant differences for a combined endpoint (all-cause death, stroke, atrioventricular block requiring permanent pacemaker implantation) between either of these three groups during a median follow-up of 779 days after TAVI. Rates of post-dilatation and paravalvular leakage where higher in the “extreme severe” AS group.
• *Karaduman et al.* [15] compared SAS (n = 371) and VSAS (n = 134) using all-cause death as primary endpoint with a follow-up of 80 months after TAVI. Total mortality was not significantly different (30.3 % vs. 24.6 %; p = 0.216), but in the Cox regression adjusted analysis patients with VSAS yielded a significant better survival rate than patients with SAS (p < 0.001). The need for pre-dilatation was higher in VSAS (84.1 % vs. 69.1 %; p = 0.001) however, procedural outcomes showed no significant differences.

**Table 4:** Overview literature: Studies comparing clinical outcomes after TAVI in SAS versus VSAS.

Study, year of publication	Design	n (SAS/VSAS)	Follow-up	Primary endpoint	Results	Procedural safety
Onoda, 2024 [13]	retrospective	174/65	3 years	all-cause death, HF-readmission	VSAS better (p = 0.01)	n. s.
Angelillis, 2023 [9]	prospective	535/102	30 days	all-cause death	not significant; readmission: VSAS better	n. s.***
Kobayashi, 2023 [10]	retrospective	281/85	1 year	MACE*	not significant	n. s.
Saji, 2021 [11]		363/258/62**	779 days	all-cause death, stroke, AVB III	not significant between all three groups	n. s.***
Karaduman, 2021 [12]	retrospective	371/134	80 months	all-cause death	not significant; adjusted: VSAS better	n. s.***
* all-cause death, stroke, HF-rehospitalization, major bleeding; ** “extremely severe” aortic stenosis (AV Vmax ≥ 6 m/s); *** pre- and/or post-dilation and/or paravalvular leakage: more frequent in more severe AS (see text)						

SAS, severe aortic valve stenosis; VSAS, very severe aortic valve stenosis; HF, heart failure; MACE, major adverse cardiovascular events; AVB, atriventricular block; AV Vmax, aortic valve maximal systolic transvalvular flow velocity; AS, aortic valve stenosis.

Thus, the overall evidence from clinical trials seems to show a consistent trend towards better outcomes of patients with VSAS compared to patients with SAS. The size of the patient groups in our study (n = 96 and n = 379 for VSAS and SAS respectively; n = 96 for both groups after matching) fits into the spectrum of the studies mentioned. In contrast to the studies mentioned above, we investigated this effect using propensity score matching. However, it would be desirable to confirm these results by prospective studies of greater statistical power.

### 4.2 Possible pathophysiological explanations

There are several putative pathophysiological explanations why the benefit from TAVI might be higher than expected in patients with VSAS:

• *VSAS as a “marker” for good left ventricular contractility:* VSAS – defined by Vmax and MPG – is inherently a marker not only for a heavily reduced systolic cross-sectional area of the aortic valve, but also for a preserved left ventricular inotropy. This contractile capacity – despite the presence of a VSAS – could contribute to a particularly favourable outcome after timely TAVI in this subgroup.
• *Possible proportion of more advanced disease in the SAS group:* On the other hand, some of the patients with “SAS” – as defined by Vmax and MPD – could also represent cases of even more advanced VSAS with preserved or mildly impaired LV-Function and relative (“paradoxical”) LFLG constellation. These would then represent a group of patients in whom the optimal time window for valve implantation had already been exceeded at the time of TAVI. This could partly explain the poorer prognosis of the SAS group. Indeed, LVEF in the lower normal range in the SAS group of our study cohort was found to be an independent predictor of poorer survival by regression analysis; however, AVC, AVCd, AVCi, and SVi did not emerge as independent predictors. In this context, it is interesting to note that NYHA class III was more prevalent in the SAS than in the VSAS group at baseline.
• *Potential independent prognostic impact of left ventricular hypertrophy:* Previous studies have emphasized the importance of left ventricular hypertrophy (LVH) and LVH regression after AV replacement, which has been shown to be more pronounced in VSAS than in SAS. [12] It has been hypothesized that this may contribute to the good prognosis in VSAS after TAVI, since the amount of LVH corresponds to clinical outcomes. [20] Complementary echocardiographic or cardiac magnetic resonance imaging measures used to assess LV systolic/diastolic function or remodeling – including strain, tissue doppler imaging, assessment of myocardial fibrosis – might also be useful but went beyond the scope of our analysis.

### 4.3 Potential clinical implications

The results of our study, which confirm existing, but preliminary evidence, may have several potential implications for clinical decision making:

• *Asymptomatic VSAS is an indication for valve replacement:* These findings – which are irrespective of symptom severity – clearly reinforce the recommendation for valve replacement in VSAS even in asymptomatic patients as given by current international guidelines. [1,2]
• *There is no reason for not performing TAVI in VSAS:* Our study confirms the results of previous studies that patients with VSAS have a particularly high benefit from valve replacement by TAVI. [[12], [13], [14], [15], [16]] Even if inherently associated with unfavourable characteristics such as more advanced symptoms, more pronounced left ventricular hypertrophy, higher calcium load, higher rates of pre- and/or postdilation and paravalvular leakage, the diagnosis of VSAS should by no means be a reason to refrain from TAVI. TAVI can be performed efficiently and safely in this patient group.
• *Should TAVI be preferred over SAVR in VSAS?* In conjunction with the results of the study by Bohbot et al. indicating that patients with VSAS have a worse prognosis after SAVR than patients with SAS [7] it could be hypothesized that TAVI is a better treatment option than SAVR in the presence of VSAS. However, for the time being, there is no argument that the presence of VSAS *per se* should be a reason to prefer TAVI over SAVR if the latter is indicated according to current guidelines. Further studies are needed to clarify this question.
• *Appropriate timing of valve replacement in asymptomatic SAS:* The results of our and other similar studies cannot be interpreted to mean that it is safe to wait until a patient progresses from SAS to the VSAS stage. The consistent, well-founded, and clinically proven recommendations of current guidelines for the indication for valve replacement in SAS should not be called into question. These are: the presence of specific symptoms, or the fulfillment of defined high-risk criteria (VSAS; progression of AS; biomarkers; aortic valve calcium load). [1,2]
Randomized controlled trials even suggest that the indications for valve replacement therapy in asymptomatic SAS patients should be extended. [21,22] These include the recently published EARLY TAVR trial, [22] which showed that TAVI in asymptomatic severe AS (AV Vmax ≥4.0 m/s; MPG ≥ 40 mmHg) was superior to clinical surveillance with respect to the composite primary endpoint (death, stroke, unplanned cardiovascular hospitalization). However, the result was driven primarily by a reduction of hospitalizations, whereas mortality and stroke did not differ significantly. Remarkably, the study did not exclude VSAS, and the subgroup analyses of SAS (AV Vmax < 5.0 m/s; n = 818) and VSAS (AV Vmax ≥ 5.0 m/s; n = 74) showed a significant benefit of TAVI versus clinical surveillance in the SAS group (HR 0.49; 95 % CI 0.38 – 062), but not in the VSAS group (HR 0.60; 95 % CI 0.29 – 1.28) in terms of the combined endpoint. However, the individual components of the combined clinical endpoint were not specified in this context, nor were the figures for the direct comparison of SAS versus VSAS, making it difficult to interpret these (supplementary) data. [[21]; supplementary appendix] Due to the results and some concerns about the design of the EARLY TAVR trial, experts commented that, for the time being, it is still reasonable to wait for the onset of symptoms in asymptomatic patients with SAS.
• *Not all SAS are the same*. Rather than using these partially contradictory findings as an argument for or against “early TAVI” in SAS, we believe the present data – as well as clinical experience – suggest that SAS is a more heterogeneous entity than previously thought. In addition to the established risk factors mentioned above, clinicians must also be aware that, for example, more advanced stages of VSAS (with a low-flow, low-gradient configuration) may appear to be “SAS” at first glance on echocardiography. It is of utmost importance to identify these high-risk groups that will benefit from rapid valve replacement therapy to prevent potentially life-threatening treatment delay. On the other hand, there are low-risk forms of AS that can certainly continue to be observed by watchful waiting without adverse consequences – e.g. truly asymptomatic, physically active patients with “less significant” SAS, in the setting of preserved ventricular functions and absence of other “red flags”.

Thus, to ensure adequate risk stratification, a broader range of parameters (imaging markers, biomarkers) may need to be considered that reflect both left heart (e.g. LVEF, SVi) and right heart function (e.g. pulmonary artery pressure, right ventricular function). These criteria should be validated in larger studies.

## 5 Limitations and strengths

Several potential limitations should be considered in this study. The design is retrospective, the cohort is of moderate size, and the data are from a single centre. Although the study cohort is relatively small, the use of rigorous propensity score matching enhances the validity of our findings by minimizing confounding and selection bias. To focus on comparing VSAS with SAS we excluded patients with an aortic valve Vmax < 4.0 m/s, which included those with low-flow low-gradient AS and those with moderate AS. While we hypothesized that cardiac reserve might play a key role in better clinical outcomes for individuals with VSAS, we did not present supporting data, such as longitudinal global strain or imaging data (MR), to confirm this. The strengths of our study lie in the comprehensive collection of electrocardiographic, procedural, and clinical data. In contrast to previous studies, we conducted a propensity score matching.

## 6 Conclusion

Our analysis confirms – in line with previous studies – several aspects: (a) Patients with VSAS represent a substantial subgroup of AS patients and achieve at least as good – possibly even better – clinical outcomes after TAVI as patients with SAS. Factors inherently associated with an advanced stage of AS, such as a higher degree of valvular calcification, higher rates of pre- and post-dilatation and PVR, do not appear to have an unfavourable effect. (b) Valve replacement therefore should not be withheld in patients with VSAS. Whether TAVI should be preferred over SAVR in these patients remains to be determined by future research. (c) It can be assumed that the group of patients with SAS is more heterogeneous than previously thought. It seems probable that there exist subpopulations with high-risk constellations that particularly benefit from urgent valve replacement. Defining criteria have to be clarified by future studies.

## CRediT authorship contribution statement

**Matthias Hammerer:** Writing – original draft, Visualization, Investigation, Conceptualization. **Elke Boxhammer:** Writing – original draft, Investigation, Formal analysis, Data curation. **Erika Prinz:** Writing – review & editing. **Bernhard Scharinger:** Investigation. **Wilfried Wintersteller:** Writing – review & editing. **Uta C. Hoppe:** Writing – review & editing.

## Declaration of competing interest

The authors declare that they have no known competing financial interests or personal relationships that could have appeared to influence the work reported in this paper.

## Data Availability

The data underlying this article will be shared on reasonable request to the corresponding author.
